# Citric Acid-Based Intrinsic Band-Shifting Photoluminescent Materials

**DOI:** 10.34133/research.0152

**Published:** 2023-05-29

**Authors:** Dingbowen Wang, Yizhu Chen, Tunan Xia, Mariana Claudino, Allison Melendez, Xingjie Ni, Cheng Dong, Zhiwen Liu, Jian Yang

**Affiliations:** ^1^ Department of Biomedical Engineering, Materials Research Institute, The Huck Institutes of the Life Sciences, The Pennsylvania State University, University Park, PA 16802, USA.; ^2^ Department of Electrical Engineering, Materials Research Institute, The Pennsylvania State University, University Park, PA 16802, USA.

## Abstract

Citric acid, an important metabolite with abundant reactive groups, has been demonstrated as a promising starting material to synthesize diverse photoluminescent materials including small molecules, polymers, and carbon dots. The unique citrate chemistry enables the development of a series of citric acid-based molecules and nanomaterials with intriguing intrinsic band-shifting behavior, where the emission wavelength shifts as the excitation wavelength increases, ideal for chromatic imaging and many other applications. In this review, we discuss the concept of “intrinsic band-shifting photoluminescent materials”, introduce the recent advances in citric acid-based intrinsic band-shifting materials, and discuss their potential applications such as chromatic imaging and multimodal sensing. It is our hope that the insightful and forward-thinking discussion in this review will spur the innovation and applications of the unique band-shifting photoluminescent materials.

## Introduction

### Citric acid-based materials

Citric acid or citrate (CA) is an essential intermediate in the tricarboxylic acid cycle (aka citric acid cycle or Krebs cycle), a central metabolic pathway for most aerobic species, and participates in a variety of substance and energy metabolism activities. Besides its crucial biological functions, CA can act as a versatile building block in the design of functional molecules and materials. Since it has 3 carboxyl groups and 1 hydroxyl group, the abundance of functional groups renders CA great flexibility to react with diverse chemicals and produce various types of chemical products such as ester, imide, urethane, anhydride, and ether. In recent years, CA-based polymers and small molecules have emerged as a novel family of materials for a large number of applications in different fields, including tissue engineering and regenerative medicine [1–11], biosensing [12–14], imaging [15–17], and antimicrobial treatment [18,19]. From a photoluminescent materials perspective, CA-based fluorophores and polymers represent an emerging class of optical materials, thereof some possessing high quantum yields (>70%), while others exhibiting intriguing photophysical properties, such as band-shifting behaviors.

### Band-shifting behavior in photoluminescent materials

Any environmental factor that influences the emission band of a material can result in band shift. Temperature, for example, is one of the common factors to cause band shift and has been harnessed for fluorometric temperature sensing or luminescence thermometry applications [20–31]. Besides temperature, many other factors, such as pH [32,33], solvent/polarity [34–41], molecular weight [42], pressure/mechanical force [43–47], and viscosity [48,49], can also cause band shift. Different material research communities lack a unified term for this emission shift phenomenon. In the organic photoluminescent materials community, “environmental factor-dependent fluorescence/emission” is the most commonly used description, probably due to the clear indication of the influencing factor of the emission band, but often times, this is unclear and confusing because the nature of the fluorescence change is not clearly stated (intensity, wavelength, phase or polarization, etc.). For example, not all the so-called environmental-factor-dependent fluorescence/emission literature are related to band shift since some of them only show environmental-factor-dependent intensity change (i.e., only the emission intensity varies with the change of certain factors) and do not change the emission wavelength or spectral line shape [50,51].

Here, we suggest adopting “band-shifting” and “band shift” to describe the phenomenon that the photoluminescence emission wavelength change with excitation wavelength and certain environmental factors, such as temperature and solvent, which can better unify the same phenomenon in different fields and eliminate ambiguity caused by existing naming confusion. Furthermore, we coined the term “intrinsic band-shifting” referring to environmental-factor-independent and excitation-wavelength-dependent emission. The intrinsic band-shifting behavior is a counterintuitive luminescent phenomenon that violates Kasha’s rule and has gained more and more attention due to the great potential in various optical applications.

### Band-shifting behavior in photoluminescent materials

Kasha’s rule states that the same fluorescence emission spectrum is generally observed irrespective of the excitation wavelength, meaning that typically the fluorescence emission spectrum of a fluorophore remains the same upon a change in the excitation wavelength [52]. The microscopic explanation of Kasha’s rule is that for most fluorophores, the emission only involves the transition from the lowest excited electronic state (S_1_) to the ground state (S_0_). Even if an electron is excited into higher electronic states, it rapidly relaxes back to S_1_ through nonradiative decay, followed by fluorescence emission and resulting in an identical emission spectrum. Exceptions that break Kasha’s rule exist. One approach is by emissions from higher excited electronic states (e.g., S_3_ to S_0_, S_2_ to S_1_). Certain molecules such as the well-known azulene, as well as thiones, pyrene, cyclazine, some of their derivatives, and other delicately designed materials have been found to possess abnormal emission due to ultrafast radiative rates or large S_2_-S_1_ energy gap [53–60]. However, these so-called anti-Kasha emissions only involve 2 or 3 electronic states, resulting in very limited emission shifts or color changes. Other mechanisms have also been exploited to achieve anti-Kasha emissions, including excited-state intramolecular proton transfer [61–65], twisted intramolecular charge transfer [66,67], adjusting pH [68,69], supramolecular self-assemblies [70–73], formation of excimers [74,75], formation of intermolecular interactions [76,77], combination of singlet and triplet excited states [78,79], copolymers [80,81], metal–organic frameworks [82,83] and covalent organic frameworks [84]. Interested readers can refer to a recent comprehensive review on anti-Kasha dual emission by Wang et al. [85] for more detailed discussions. Furthermore, band shift or anti-Kasha emission has also been reported in ultralong (organic) room-temperature phosphorescence materials [86–89], a new class of phosphorescent materials that emerged in this decade. Unfortunately, most of the aforementioned materials share similar drawbacks as higher-excited-state emissive fluorophores, such as azulene: only limited emission shifts can be realized, and their anomalous emissions are usually called “dual-emission”, indicating that only 2 potential emission modes can be harnessed. This is unfavorable for optical applications requiring large and continuous or multiple emission shifts, such as chromatic imaging [90–92]. The abovementioned intrinsic band-shifting materials are promising for such optical applications without changing the working conditions of these materials. The current organic photoluminescent materials showing continuous or multiple intrinsic band-shifting behaviors can be categorized as small-molecule fluorophores, fluorescent polymers, and carbon dots (CDs).

### Purposes and focus of this review

In stark contrast to its intriguing photophysical properties and broad application prospects, a systematic review is currently lacking on band-shifting photoluminescent materials. Therefore, like a Chinese old saying, “throwing bricks to attract jade”. In this review, we aim to discuss on the band-shifting photoluminescent materials with a focus on CA-based intrinsic band-shifting photoluminescent materials with intrinsic band-shifting behaviors (i.e., excitation-wavelength-dependent emission spectra under a constant environment). We also discuss their potential applications, limitations, and outlook of their future directions of development. It is our hope that the insightful and forward-thinking discussion in this review will spur the innovation and applications of the unique band-shifting photoluminescent materials.

## Citric Acid-Based Intrinsic Band-Shifting Photoluminescent Materials

### CA-based fluorophores and fluorescent polymers with intrinsic band-shifting behaviors

Since the unexpected fluorescence property of the product from the condensation reaction between CA and amino acids was first demonstrated by our group in 2009 [16], a number of related works have been reported [12,13,15,93–104]. The initial work started with an occasional observation of blue fluorescence emitted from the condensation product of CA, L-cysteine, and 1,8-octanediol under strong summer sunlight. This was intriguing at the time since it was the first time that photoluminescent biodegradable polymers could be produced without introducing traditional conjugated organic dyes [16]. In addition, we found that CA could react with any amine-containing molecules including α-amino acids, β-amino acids, and primary amines through convenient condensation reactions to generate fluorescent molecules [15,16,96,99]. The versatile CA chemistry enabled the discovery of 2 classes of CA-based fluorophore structures: thiazolopyridine carboxylic acid structures (TPAs) and dioxo-pyridine ring structures (DPRs) [15]. The TPAs present strong excitation-wavelength-independent fluorescence emissions, while the DPRs exhibit interesting excitation-wavelength-dependent emission or band-shifting behavior. The emission peaks of DPR fluorophores gradually shifts to longer/redder wavelengths as the excitation wavelength increases. This continuous intrinsic band-shifting behavior is rarely reported in other material systems [105,106].

**Fig. 1.**
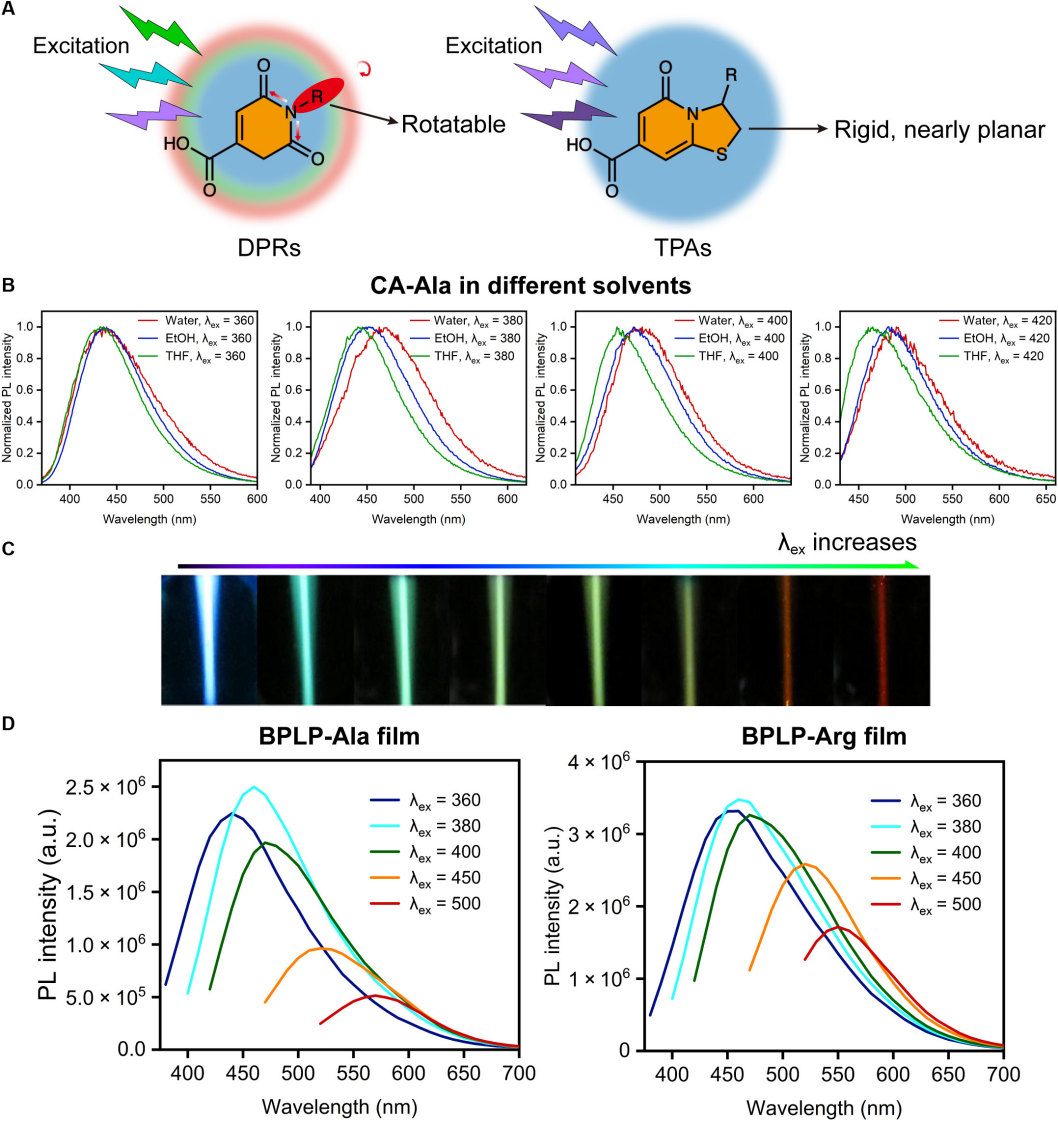
Demonstration of intrinsic band-shifting behavior (excitation-wavelength-dependent emission) of CA-based fluorophores and polymers. (A) Emissive mechanisms and structural comparison of DPRs and TPAs. (B) Emission spectra of CA-Ala in different solvents at various wavelengths of excitation, showing solvent polarity-dependent intrinsic band-shifting behavior. (C) Optical images of excitation-wavelength-dependent emission of CA-Ala solution. From left to right side, the excitation wavelength increases. Adapted with permission from [15]. Copyright (2017) Elsevier. (D) Excitation-wavelength-dependent emission spectra of BPLP-Ala and BPLP-Arg films. PL, photoluminescent; a.u., arbitrary units.

**Fig. 2.**
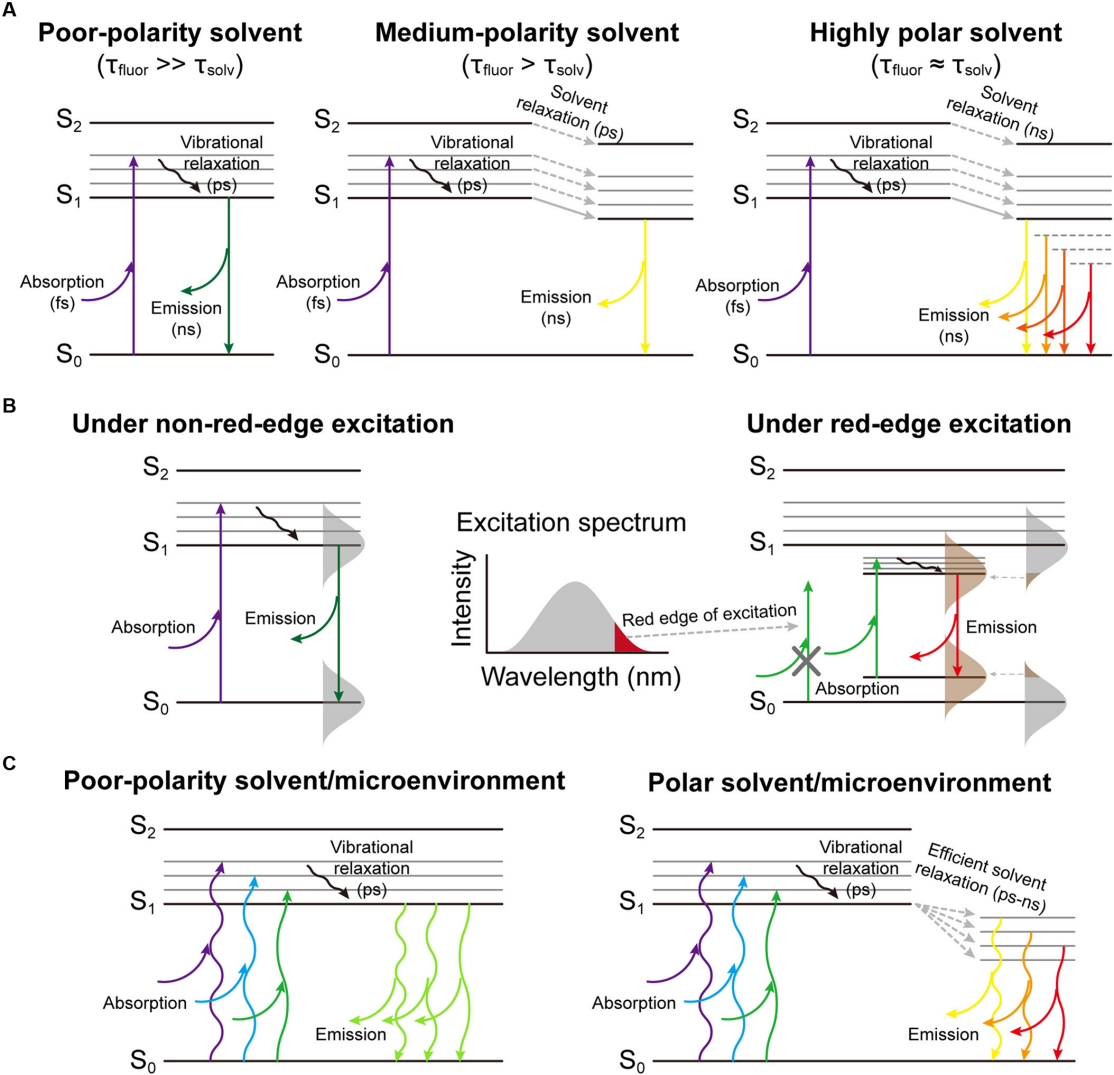
The mechanistic explanations of emission shifts. (A) The solvent polarity effects on emission shift. As the solvent polarity increases, the solvent relaxation is enhanced, resulting in a stronger emission shift. (B) Schematic illustration of REE. Under red-edge excitation, the photon energy is only sufficient to excite partial ensemble of the fluorescent species that possess narrower band gaps. With the slow rate of dipolar/dielectric relaxation, it exhibits excitation-wavelength-dependent emission. (C) The proposed mechanisms of the intrinsic band-shifting phenomenon. In a polar solvent or microenvironment, different excitation wavelength corelates to one optimal transition from the corresponding low-lying excited energy level (band) generated from solvent relaxation to the ground state, resulting in continuous intrinsic band-shifting phenomenon. τ_fluo_ denotes fluorescence lifetime or excited-state lifetime, and τ_solv_ denotes solvent relaxation time.

The excitation-wavelength-dependent emission in certain fixed media or conditions has been reported for decades [107–114], whereas the mechanisms are still under debating. It was proposed that the fluorescence of DPRs is attributed to the tertiary nitrogen of its imide structure, as previously suggested for hyperbranched poly (amido amine) dendrimers [15,115,116]. More specifically, the adjacent carbonyl groups not only extend the resonance of the tertiary nitrogen but also redshift its n-π* and n-σ* transitions of the lone pair electrons due to the electron-withdrawing effects, and therefore result in a moderate fluorescence in the visible range (Fig. 1A). Take a DPR type of intrinsic band-shifting small molecular fluorophore, CA-Ala, as an example; the time-resolved fluorescence data shows that the fluorescence decay of CA-Ala cannot be fitted to either single- or double-exponential decay, indicating the emission is not from a single energy band but multiple excited-state energy levels [15]. The extent of the band shift is shown to increase with solvent polarity (Fig. 1B and Table 1), and lifetimes vary markedly in highly polar solvents, such as water, but remain relatively constant in poor-polarity solvents [15]. All of the DPRs possess pendant rotatable groups attached to the tertiary amine nitrogen that are mostly polar and can interact with surrounding molecules, while in TPA type of fluorophores, the fused 5-membered ring strongly confines the polar groups into a nearly planar conformation, resulting in no band-shifting behavior (Fig. 1A). An optical image of CA-derived biodegradable photoluminescent polymer-alanine (BPLP-Ala) solution under different wavelengths of excitation is given in Fig. 1C to show the continuous band shifts. Furthermore, we have also found that thermally cross-linked BPLP films exhibit an even more enhanced band-shifting phenomenon (Fig. 1D). These results indicate that the solvent or environmental polarity could significantly affect the relaxation kinetics of DPRs and further change the emission properties of DPRs. Three Jablonski diagrams are presented in Fig. 2A to illustrate the solvent effects: in a nonpolar or poor-polarity solvent, negligible solvent relaxation exists due to the extremely weak dipole interactions between the fluorophore and the surrounding solvent molecules, and therefore, no redshift of emission can be observed; in a moderate-polarity solvent, a solvent relaxation process, which is on the picosecond scale and faster than the nanosecond-scale emission process, occurs and causes a constant redshift of the emission; in a high-polarity solvent, the strong dipole interactions efficiently prolong the solvent relaxation time to nanosecond scale, which is comparable to the emission time scale, resulting in a series of emissive low-lying excited states, a broader emission spectrum, and a multiexponential fluorescence temporal decay. Nevertheless, the solvent effect can only explain the emission spectrum broadening and solvent-dependent emission shift; but the intrinsic band-shifting phenomenon or the excitation-wavelength-dependent emission remains a mystery. As shown in Fig. 2B, the excitation-wavelength-dependent emission or band-shifting behavior of DPRs can be partially explained by “red-edge effects” (REE), which states that the excitation-dependent-emission originates from the fluorophore-environment interactions (dipole interactions) [117–120]. The readers who want to get a more in-depth understanding of the REE mechanistic explanation are recommended to read the comprehensive review by Demchenko [117]. Based on the explanation therein, in condensed media, the energy of any ground- or excited-state level, *E*, can be expressed as:

E=E0+ΩWdd (1)

where *E*_0_ denotes the pristine energy of the molecule, and Ω(*W*_dd_) is the summation of the so-called solvation or stabilization energy (*W*_dd_) from the elementary solute–solvent interactions in the ensemble, the most important of which are dipole–dipole interactions. There are 2 types of distributions for ground state (Ω(*W*_dd_^g^)) and excited state (Ω(*W*_dd_^e^)), respectively, due to the different dipole moment (μ) of the fluorophore in the corresponding state. When the excitation energy is high enough (relatively short excitation wavelength), i.e., the mean excitation energy is sufficient to exceed the mean band gap (*h*ν^mean^ ≥ *E*_e_^mean^ − *E*_g_^mean^), the emission is excitation-wavelength-independent and there is no band shift. If the excitation energy is so low (reaching the red edge of the absorption spectrum) that it cannot excite all members of the ensemble, only the fluorophores constituting a part of the distribution (from the lower part of the distribution Ω(*W*_dd_^e^) coming from the species that interact most strongly with the environment in the excited state to the upper part of the distribution Ω(*W*_dd_^g^) originating from the species that interact the least strongly with the environment in the ground state) can then be selectively excited. As a result, the emission shifts to the longer wavelength as the excitation wavelength increases within the red edge. However, REE theory cannot fully interpret the band-shifting phenomenon because first and foremost, the excitation-wavelength-dependent emission of CA-derived intrinsic band-shifting fluorophores and polymers is not just confined within the red edge of the excitation/absorption spectrum but also extends to relatively short excitation wavelength and even below the absorption peak wavelength. Second, the REE emphasizes the key role of frozen or relatively-slow-structural-dynamics medium, from vitrified and highly viscous solutions to polymer matrices, on the emission shift since they slow down the redistribution of the fluorophore and prolong the solvent dipolar/dielectric relaxation, resulting in the REE [117,121]. Once the solvent becomes low-viscous, the REE becomes undetected, but the band-shifting behavior of CA-derived intrinsic band-shifting fluorophores and polymers can still be observed in low-viscosity solvents, such as water, at room temperature. Thus, we hypothesize that the primary mechanisms of the band-shifting behaviors of CA-based photoluminescent materials should still correlate to the fluorophore-environment interactions, especially the dipole interactions. In a nonpolar solvent, the dipole interactions are negligible resulting in excitation-wavelength-independent emission, while as solvent polarity increases, the fluorophore-environment interactions become more and more appreciable. These interactions generate a series of low-lying energy bands, whereas the oscillator strength of transitions between different bands depends on the excitation wavelength, therefore causing a continuous band shift or excitation-wavelength-dependent emission (Fig. 2C) and supported by the experimental results (Fig. 1B and Table 1). Since the REE and the mechanisms proposed above can also be applied to condensed media including solid-phase conditions, the band-shifting behavior of BPLP films is not a total surprise. We propose that 1,8-octanediol works as a linker or spacer to separate the molecular fluorophores and the concentration of the fluorophores is not too high in our BPLP polymers, both of which facilitate the formation of a sol-gel-like ensemble and enable the dipole interactions between polar pendant groups and the surrounding microenvironment.

**Table 1.** Emission maximum of CA-Ala at various excitation wavelength in different solvents with diverse polarity.

Solvent	Snyder polarity [212]	Reich polarity [213]	λ_ex_ (nm)	Em_max_ (nm)
Water	9.0	1.0	360	439
			380	463
			400	481
			420	490
Ethanol (EtOH)	5.2	0.654	360	436
			380	452
			400	472
			420	482
Tetrahydrofuran (THF)	4.2	0.207	360	433
			380	445
			400	454
			420	465

**Fig. 3.**
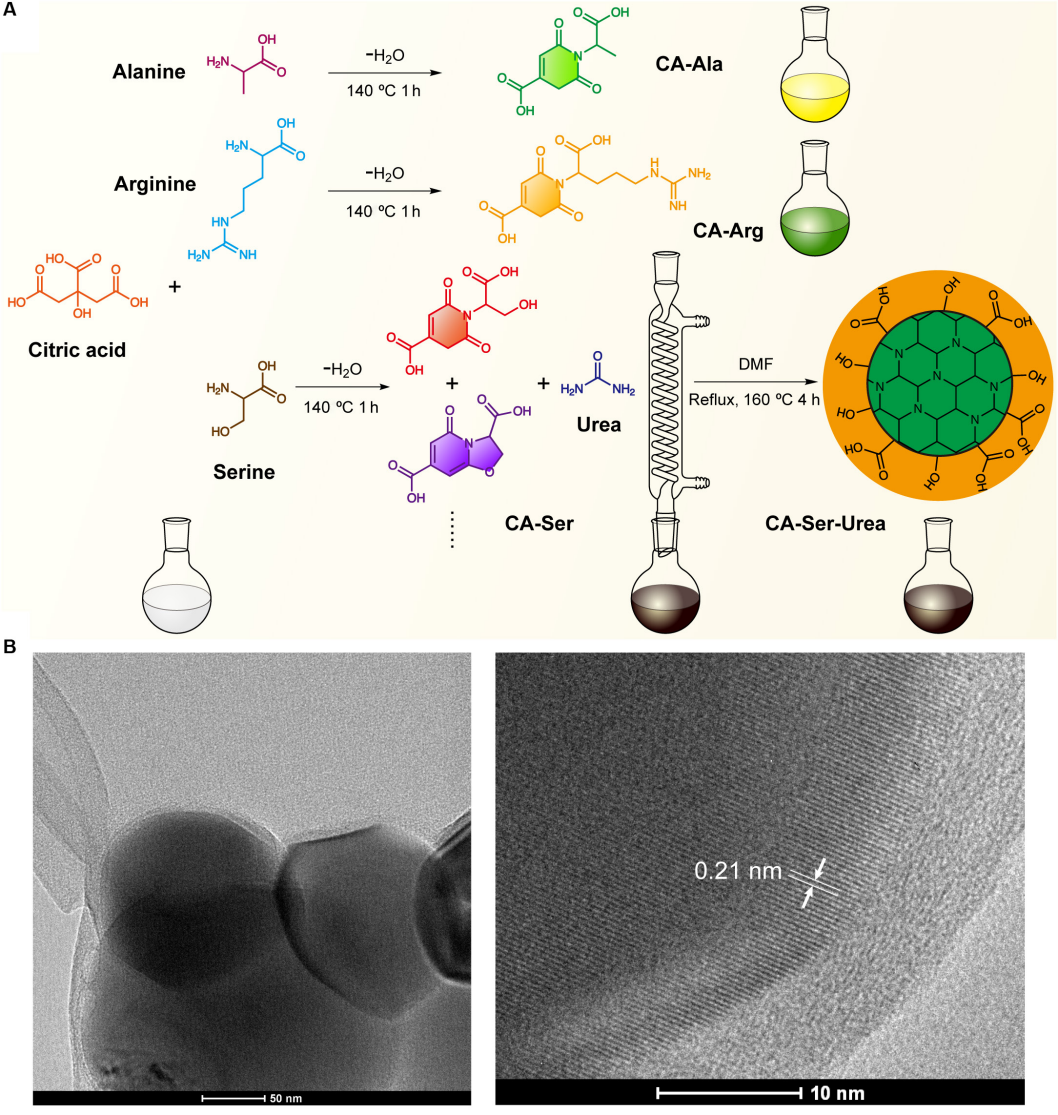
The synthetic route of CA-Ala, CA-Arg, CA-Ser, and CA-Ser-Urea and morphology of CA-Ser-Urea. (A) The 1-pot condensation reaction synthesis of CA-Ala, CA-Arg, and CA-Ser from CA and amino acids and the solvothermal synthesis of CA-Ser-Urea from CA-Ser and urea. (B) The TEM images of CA-Ser-Urea. The high-resolution TEM image shows visible crystalline lattice fringes with a 0.21-nm interlayer spacing. DMF, N,N-dimethylformamide.

CA-Arg, CA-Ala, and CA-Ser (which are small molecular fluorophores [15]) and a novel CA-based nanomaterial CA-Ser-Urea are chosen in this paper as examples of CA-derived intrinsic band-shift materials. The synthesis of CA-based fluorophores, which has been reported in our previous publications, was via a 1-pot reaction of CA and a primary amine-containing compound in water or solvent-free conditions [15]. The CA-Arg and CA-Ala are typical dioxopyridine (DPR) type of chromophores, as shown in Fig. 3A, whereas the dark brown color of CA-Ser implies its heterogeneity of composition since a single type of DPR molecules cannot possess such a broad absorption spectrum. Because serine has a reactive hydroxyl group that could work as a linker to CA carboxyls, the possible composition of CA-Ser is a mixture/entangled polymer network of 2 molecular chromophores (Fig. 3A) interconnected by CA esters and serine esters or plus further reaction, which is assigned to a form of nonenzymatic browning but different from Maillard reaction. As for CA-Ser-Urea, which is first reported in this work, it is considered to be a carbon nanomaterial and is to be discussed in the following sections.

### CA-based CDs with excitation-wavelength-dependent emission

CDs or carbon nanodots (CNDs) have emerged as an intriguing and novel class of photoluminescent materials and have shown extraordinary potential in many fields and application scenarios such as sensing, imaging, display, anticounterfeiting, and information encryption, due to their low-cost and facile hydrothermal/solvothermal or pyrolysis synthesis, excellent photophysical tunability, and good biocompatibility [122–126]. With sizes below 10 nm, CDs exhibit promising optical properties, especially excellent photoluminescent properties [127]. Interestingly, some raw materials combination and synthetic routes yield excitation-wavelength-dependent emission [128–140]. The mechanisms underlying this intriguing phenomenon are still unresolved. The mainstream explanations include quantum-confinement effect (also known as size effect) [136,141–159], surface traps [136,140,144,147,160 –173], giant REE [174], edge states [175–178], electronegativity of heteroatoms model [179–182], and other mechanisms [183]. The most prevalent theory ascribes it to dot-dot variation or quantum-confinement effect, meaning that there are different CDs with tiny size differences or diverse electronic states in the final products, but the fluorescence of every single dot is excitation-wavelength-independent. Li et al. [148] highlighted that the quantum-confinement effects dominate the optical properties of CDs and the emission is size-dependent photoluminescence. Wang et al. [134] emphasized that the “edge states” on or close to the surface of CNDs including carboxyl and carbonyl groups could be nonradiative states competing with the emission center and working as so-called “traps”, further dominating the optical properties of the CNDs. Sharma et al. [132] also stated that different types of aggregates of CDs even at very dilute solution generate multiple discrete electronic states leading to significant excitation-wavelength-dependent emission, where surface-exposed functional groups play a crucial role in determining the extent and nature of aggregation and thus excitation-wavelength-dependent emission spectra. Ding et al. [184] also demonstrated that by using column chromatography, different types of CDs with excitation-wavelength-independent emission and various surface oxidation states could be separated. However, some others questioned this explanation and brought up other theories. For example, Dam et al. [129] reported single dots with excitation-wavelength-dependent emission property and confirmed the emission was coming from a single dot through detailed characterizations. Pan et al. [128] reported excitation-wavelength-dependent emissive CDs, precluded the possible origin of the band shifting from multiple dots by using single-particle fluorescence imaging, and speculated that the band-shifting behavior originates from C-N/C-O- and/or C-N-related functional groups. Fu et al. [133] postulated that the excitation-wavelength-dependent emission was attributed to a simultaneous energy transfer from polycyclic aromatic hydrocarbon cores with larger energy gaps to those with smaller energy gaps. It is worth noting that the work of Mishra et al. [185] drew our attention since they pointed out that with appropriate and extremely careful purification, every single fraction of CDs shows excitation-wavelength-independent emission, and the actual origin of excitation-wavelength-dependent emission and spectral migration lies with its ground-state optical heterogeneity. They also claimed that the putative molecular impurity misled CDs literature. In general, the emissive and spectral migration mechanisms of CDs are still in debate and need further studies. There is also a comprehensive review that summarizes the photoluminescent mechanisms of different CDs including graphene quantum dots, CNDs, and polymer dots [186].

Amid myriads of CDs starting materials, CA is one of the most promising compounds to make CDs, as CA possesses 3 carboxyl groups and 1 hydroxyl group rendering it great potential and capability to react with other compounds as an excellent carbon source. The evidence is that CA-based CDs account for a large proportion of the aforementioned CDs-related works, and some representative CDs synthesized from CA are listed in Table 2. To achieve efficient fluorescence emission in CA-based fluorophores, the introduction of N atom(s) is usually exploited. Urea is one of the most commonly applied amines to synthesize CA-based CDs [185,187–195], owing to its small molecular weight, plural functional groups, and high N content. Among these CA-Urea-based CDs, only a few show excitation-wavelength-dependent emission properties. Inspired by this, we chose CA-Ser, a fluorophore that is synthesized from CA and L-serine and exhibits the highest quantum yield (26.02% ± 0.22% at 365-nm excitation) among the family of band-shifting DPR fluorophores, to react with urea solvothermally affording CA-Ser-Urea. The CA-Ser-Urea was synthesized by dissolving 0.5 g of CA-Ser and 1.0 g of urea into 5 ml of N,N-dimethylformamide in a 50-ml round-bottom flask. The reaction was conducted at 160 °C with stirring and reflux for 4 h (Fig. 3B). The resultant dark brown solution was collected for further tests. The morphology of CA-Ser-Urea was characterized using transmission electron microscopy (TEM). TEM images of CA-Ser-Urea show that their diameters range from 150 to 250 nm, which is much bigger than conventional CDs (usually smaller than 10 nm) and might be due to the high degree of crosslinking structure of the starting material, CA-Ser. The high-resolution TEM image shows clear crystalline lattice fringes with a 0.21 nm of interlayer spacing, which corresponds to the d-spacing of the graphene {1-100} planes [187,196] and implies that CA-Ser-Urea possesses a graphene-like carbon core (Fig. 3B). Though carbon-based nanomaterials with only 1 dimension less than 10 nm can also be called CDs, in consideration of its flake/sheet morphology and relatively large 2-dimensional size, it is referred below as CA-Ser-Urea carbon (nano)flakes.

**Table 2.** CDs synthesized from CA.

Starting materials	Synthesis method	Band shifting (Y/N)	Reference
CA, urea	Autoclave–microwave, melted, solvent free	N	[122]
CA, urea	Microwave, open/sealed vessel, water	Y (gCDin)	[123]
CA	Microwave-assisted solvothermal	Y	[128]
CA, EDA	Hydrothermal	Y (UVCDs)	[130]
CA, MEA	Hydrothermal	Y	[131]
CA, urea/DAP	Heating mantle, solvent free	Y	[132]
CA, urea	Microwave, open vessel, water	Y	[134]
CA, urea	Microwave, open vessel, water	Y	[135]
CA, EDA	Hydrothermal	Y	[136]
CA, EA	Pyrolysis	Y	[137]
CA, urea	Hydrothermal	N	[185]
CA, EDA	Microwave, open vessel, water	Y	[139]
CA, LPEI	Autoclave, water	Y	[140]

EDA, ethylenediamine; MEA, monoethanolamine; DAP, diaminopropane; EA, ethanolamine; LPEI, linear polyethyleneimine

### One-photon excited fluorescence of representative CA series of intrinsic band-shifting fluorophores and CA-Ser-Urea carbon nanoflakes

The emission wavelength of all current intrinsic band-shifting materials increases with the increase of excitation wavelength (positive band shift), to the best of our knowledge. It is indicated that as the excitation wavelengths redshift, the corresponding optimal emission state also shifts to lower energy states. To give readers a concrete impression, the general photophysical (linear optical) properties or 1-photon excited fluorescence of intrinsic band-shifting materials are discussed below by taking the aforementioned several CA-based photoluminescent materials as typical representatives.

**Fig. 4.**
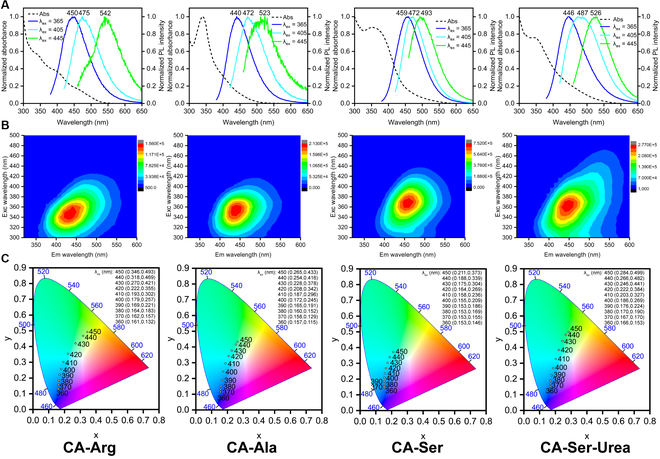
One-photon related optical property characterizations of CA-Arg, CA-Ala, CA-Ser, and CA-Ser-Urea. (A) Absorption (dash line) and emission spectra under 365-, 405-, and 445-nm excitations. (B) Excitation-emission-intensity contour maps. (C) The CIE 1931 chromaticity diagrams with coordinates under different excitation wavelengths.

The 1-photon excited fluorescence spectra of the CA-based intrinsic band-shifting materials are shown in Fig. 4A and B. A noticeable bathochromic shift (redshift) of emission spectrum with an increase of excitation wavelength is observed for all materials. Moreover, the excitation-emission-intensity contour maps all exhibit oblique-elliptical-shaped distributions (Fig. 4B), which illustrate the excitation-wavelength-dependent emissions and are distinguishable from the more round-shaped or non-oblique-elliptical-shaped distribution of conventional fluorophores. In detail, the emission peak wavelength (Em_max_) of CA-Arg under the excitation wavelengths of 365, 405 and 445 nm changes from 450 to 475 and eventually to 542 nm (25-nm and 67-nm redshift). CA-Ala shows 440- to 472- to 523-nm Em_max_ (32- and 51-nm redshift, respectively) under 365-, 405-, and 445-nm excitation. CA-Ser exhibits 459- to 472- to 493-nm Em_max_, which gives 13- and 21-nm redshift and has the smallest redshift among these CA-based band-shifting materials. CA-Ser-Urea possesses 446- to 487- to 526-nm Em_max_ (41 and 39 nm) under 365-, 405-, and 445-nm excitation (Fig. 4A). The contour maps in Fig. 4B give a clear visualization of the band-shifting profile of CA-Arg, CA-Ala, CA-Ser, and CA-Ser-Urea. The y-axis represents the excitation wavelength while the x-axis represents the emission wavelength, and the color from red to blue represents the intensity from high to low. Thus, by drawing a horizontal line with a certain y intercept, an emission intensity spectrum under a specified excitation wavelength can be obtained, and the point whose color is closest to red (i.e., maximal intensity) provides the Em_max_ at this excitation wavelength. To better visualize the redshift and color change, the CIE (Commission Internationale de l'Éclairage) 1931 coordinates of CA-Arg, CA-Ala, CA-Ser, and CA-Ser-Urea were calculated and shown in Fig. 4C. Matched to the aforementioned results, CA-Ser exhibits the smallest redshift, while CA-Arg, CA-Ala and CA-Ser-Urea show a larger redshift.

As we discussed in CA-based fluorophores and fluorescent polymers with intrinsic band-shifting behaviors, the redshift or band-shifting behavior of CA-Arg and CA-Ala can be partially explained by the REEs and better explained by our hypothetical mechanisms, which is attributed to the interactions between fluorophores and the surrounding solvent molecules. We hypothesize that the presence of rotating polar group(s), such as the carboxyl group of both CA-Arg and CA-Ala and the guanidino group of CA-Arg, generates additional dipole interactions with surrounding water molecules, prolonging the solvation time to a scale comparable to the emission, further relaxes the excited state to various lower energy levels depending on the excitation wavelength and results in the band-shifting behavior. For CA-Ser, the noticeable dark-brown color indicates more than 1 chromophore with distinct absorption peaks exist. Thus, a possible origin of the excitation-wavelength-dependent emission from CA-Ser solution is the heterogeneity of the components or the presence of multiple emission centers. This can also explain the weak excitation-wavelength dependence of CA-Ser because the emission shifts only involve the chemical structures of different emissive components, which exhibit similar Em_max_, rather than a series of low-lying energy bands. Considering the sheet/flake morphology of CA-Ser-Urea, its excitation-wavelength-dependent emission could be partially explained by the “giant red-edge effects”, which is used to elucidate the origin of excitation-wavelength-dependent fluorescence emission of graphene oxide sheet. As the name suggests, the “giant red-edge effects” (giant REE) is also originated from the slow solvent relaxation in polar solvents but is applied to a larger scale of the local environment (i.e., REE is concerned with a single molecule and its surrounding solvent molecules, while giant REE features a local environment of nano-network such as graphene oxide sheet). Similar to small molecular intrinsic band-shifting fluorophores, considering that the band-shifting behavior of CA-Ser-Urea can occur in low-viscosity solvents and at relatively short excitation wavelength, our hypothesized mechanisms might be a more plausible explanation. Since the intrinsic band-shifting mechanisms behind various types of photoluminescent materials are still not fully elucidated, more investigations are needed to fully develop the theory of the excitation-wavelength-dependent fluorescence emission.

For common fluorophores, the quantum yield (Φ) exhibits little difference under different excitation wavelengths since only S_1_ to S_0_ transition is involved. For our CA-based intrinsic band-shifting materials, owing to the excitation-wavelength-dependent emission, the quantum yield (Φ) varies with the excitation wavelength. As shown in Table 3, the absolute quantum yield under 365-nm excitation (Φ_365_) of CA-Arg is 9.84%, while the quantum yield under 405-nm excitation (Φ_405_) decreases to 2.9% and the quantum yield under 445-nm excitation (Φ_445_) drops to 0.6%. CA-Ala shows a similar trend with Φ_365_ = 8.31%, Φ_405_ = 1.5%, and Φ_445_ = 0.25%. CA-Ser possesses Φ_365_ = 26.02%, Φ_405_ = 10.82%, and Φ_445_ = 1.36%, which are the highest under 365- and 405-nm excitation among the CA-based band-shifting materials studied thus far. CA-Ser-Urea exhibits the second highest quantum yield under 365- and 405-nm excitation and the highest quantum yield under 445-nm excitation (Φ_365_ = 13.85%, Φ_405_ = 6.55%, and Φ_445_ = 2.52%) and from 365- to 405- and further to 445-nm excitation, CA-Ser-Urea exhibits the slowest decrease in the quantum yield, which might be more suitable for imaging applications.

**Table 3.** Quantum yields of CA-based band shifting fluorophores at various 1-photon excitation wavelengths. The quantum yield (Φ) measurement under 365-nm excitation was carried out on a Horiba FluoroMax-4 equipped with a Quanta-φ F-3029 integrating sphere. Then, Φ under 405- and 445-nm excitation was calculated based on the ratio of area integration under the emission spectrum curves.

Fluorophores	Solvent	λ_ex_ (nm)	Quantum yield, Φ (%)
CA-Arg	Water	365	9.84 ± 0.40
		405	~2.9
		445	~0.6
CA-Ala	Water	365	8.31 ± 0.28
		405	~1.5
		445	~0.25
CA-Ser	Water	365	26.02 ± 0.2
		405	~10.82
		445	~1.36
CA-Ser-Urea	Water	365	13.85 ± 0.23
		405	~6.55
		445	~2.52

To sum up, under 1-photon excitation among the CA-based intrinsic band-shifting materials introduced thus far, CA-Arg and CA-Ala show promising redshift but lower quantum yields and the quantum yield decreases significantly as the excitation wavelength increases; CA-Ser has the highest quantum yield but the smallest redshift; CA-Ser-Urea exhibits the second highest quantum yield combining with considerable and more evenly distributed redshift, showing that CA-Ser-Urea is the best band-shifting material among these CA-based materials. For these intrinsic band-shifting photoluminescent materials, since the emission gap narrows down as the excitation wavelength increases, the oscillator strengths of the corresponding transitions decrease while nonradiative decay rates become more and more dominant, therefore resulting in the quantum yield dropping. In other words, the current intrinsic band-shifting photoluminescent materials exhibit positive band-shifting with reduced quantum yields. Moreover, an oblique- elliptical-shaped distribution of the excitation-emission-intensity contour map is a characteristic feature of intrinsic band-shifting photoluminescent materials since the maximum emission wavelength increases with the excitation wavelength.

### Two-photon excited fluorescence properties of CA-Ser-Urea nanoflakes

**Fig. 5.**
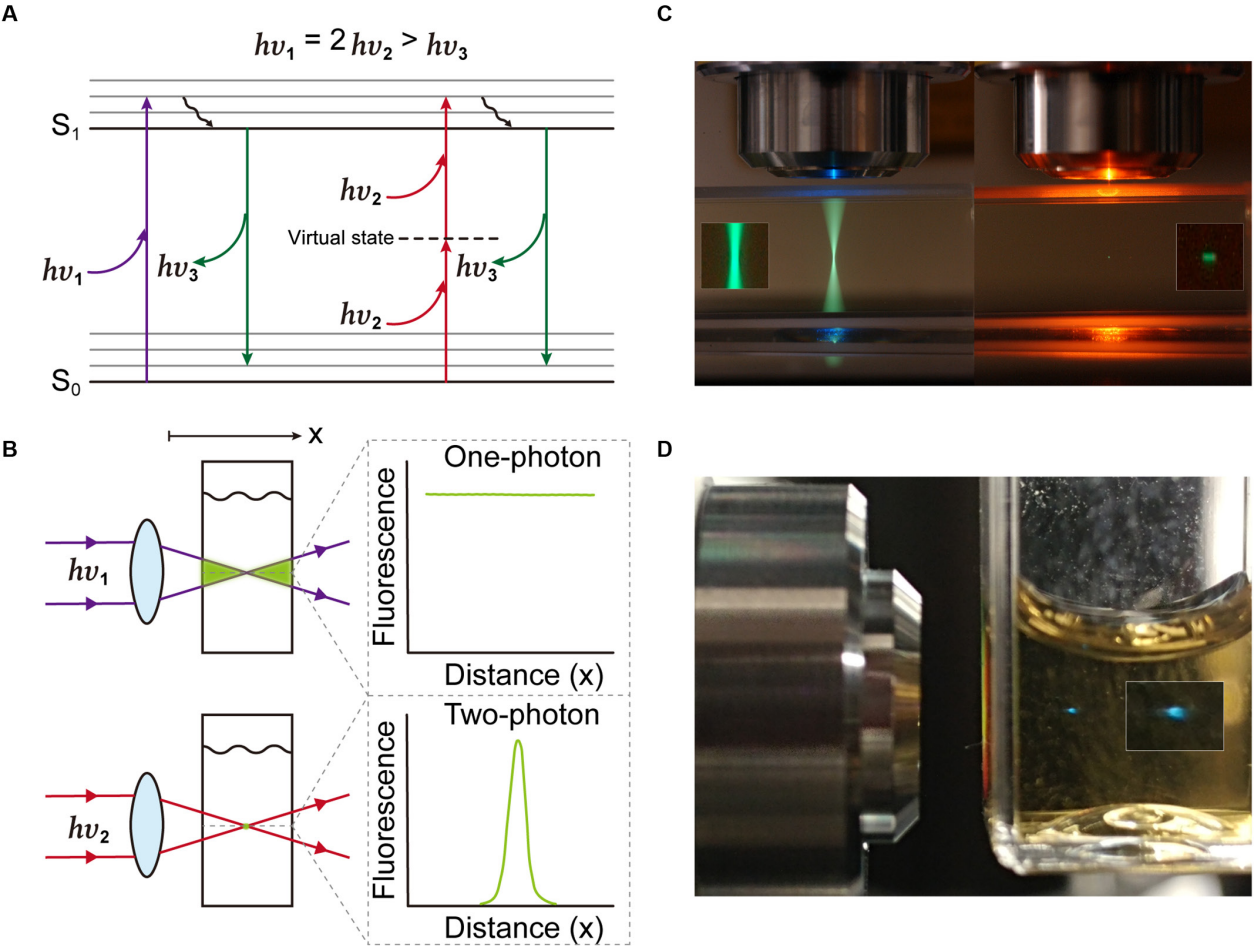
2PEF vs 1PEF. (A) A Jablonski diagram for 2PEF and 1PEF. (B) Schematic comparison of 2P and 1P excitation. (C) Real-world comparison of 2P and 1P excitation. The cuvette was filled with a fluorescein solution. The whole light path exhibits bright emission under 1P excitation (left), while confined 2P excitation results in a small-dot area of emission (right). Reprinted with permission from Dr. Steve Ruzin and Ms. Holly Aaron, UC Berkeley. (D) An image of 2PFE from CA-Ser solution (under focused 800-nm 100-fs pulsed laser excitation).

The 2-photon absorption (2PA) concept was first theoretically proposed by Maria Göppert Mayer in the 1930s [197] and was demonstrated experimentally in 1961 [198], right after the invention of the laser. It is worth mentioning that the unit of 2-photon absorption cross-section (2PCS), GM (1 GM ≡ 10^−50^ cm^4^s/photon), is named after Maria Göppert Mayer. The 2PA involves the simultaneous absorption of 2 half-energy (i.e., double wavelength or half frequency) photons, comparable in total energy to a single photon in 1-photon absorption. Figure 5A shows a Jablonski diagram illustrating the typical 1-photon excited fluorescence (1PEF) and 2-photon excited fluorescence (2PEF) processes. In a single-photon process, an electron is promoted to the excited state upon the absorption of a single photon. An electron can also be pumped into the excited states via a nearly simultaneous absorption of 2 half-energy photons mediated by a virtual state. High excitation energy density is required by 2PEF since the 2PA is a nonlinear process and the efficiency of 2PA or 2PEF is proportional to the excitation laser intensity to the second power (i.e., 2PA, 2PEF ∝ *I*^2^) [199]. As a result, the excitation is confined to the focal region, where the excitation intensity is the highest. The 2PEF is attractive for optical imaging because (a) biological tissues exhibit relatively small absorption and scattering when applying near-infrared (NIR) light; (b) the shorter wavelength radiation, especially ultraviolet (UV)/violet/blue light usually causes photodamage or phototoxicity to biological samples; (c) the parasitic emission and undesired photochemical reactions out of the focal volume are significantly reduced due to the spatially confined excitation [199–204]. Figure 5B and C provides schematic and realistic illustrations of the comparison between 1PEF and 2PEF, respectively. Imagine a 1P laser beam incident on a cuvette containing a sufficiently dilute fluorophore solution; the light absorption in any plane is approximately constant as the power distribution in each plane is about the same regardless of focusing or not, while in 2P case because the amount of light absorption is proportional to the square of the intensity, focusing the beam not only decreases the focal size but also increases the intensity, showing the highest 2PA at the focal point and a rapid drop along the axial direction [199].

**Fig. 6.**
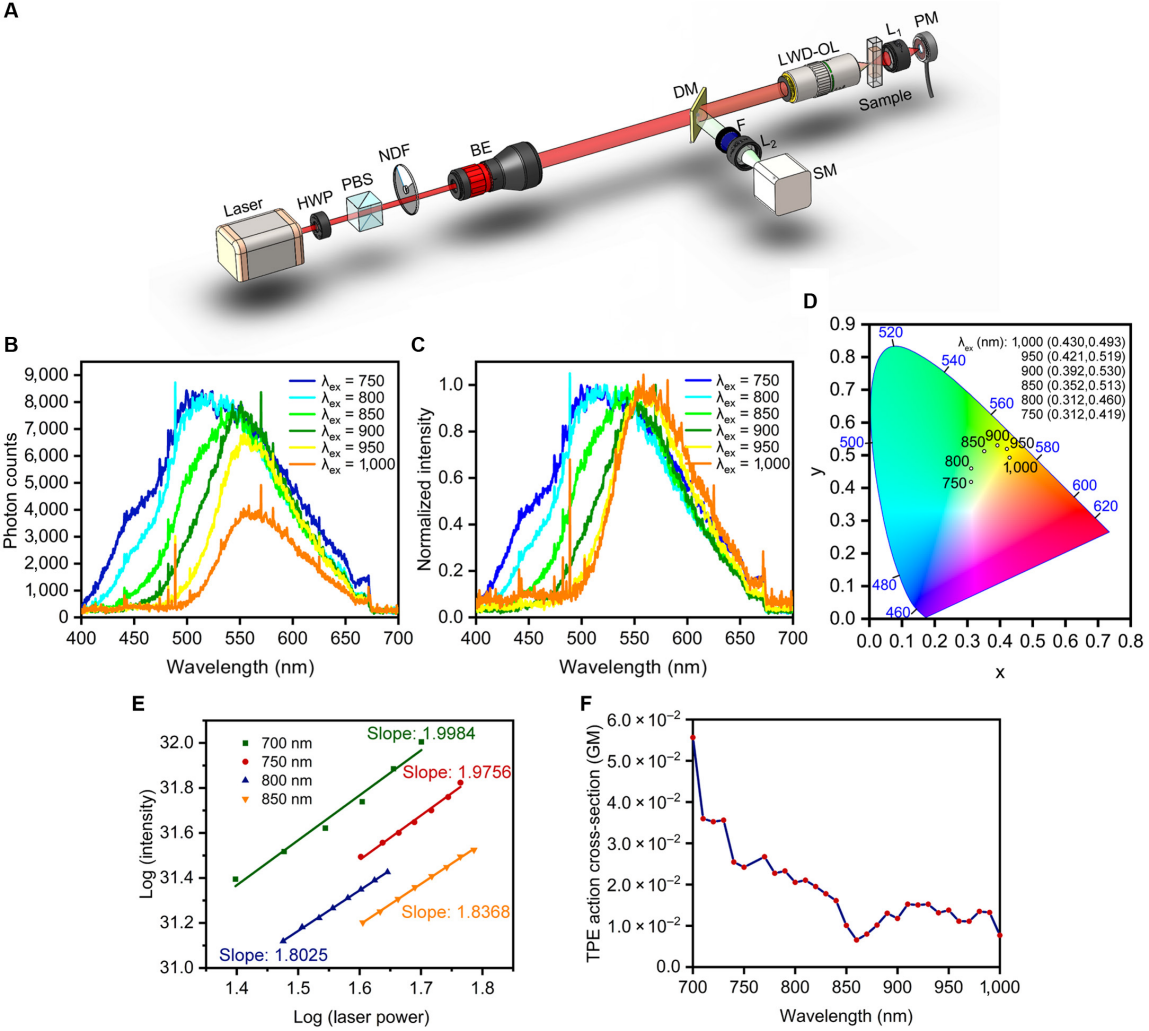
Two-photon characterizations of CA-Ser-Urea. (A) Schematic of the experimental setup (HWP, half-wave plate; PBS, polarized beam splitter; NDF, neutral density filter; BE, beam expander; DM, dichroic mirror; F, filter; L, lens; SM, spectrometer; LWD-OL, long working distance objective lens; PM, power meter). (B) 2PEF emission spectra under 750-, 800-, 850-, 900-, 950-, and 1,000-nm 2P excitations. (C) Normalized 2PEF emission spectra under 750-, 800-, 850-, 900-, 950-, and 1,000-nm 2P excitations. (D)The CIE 1931 chromaticity diagrams with coordinates under different 2P excitation wavelengths. (E) Plot of emission intensity vs excitation laser power in logarithmic scale. (F) Plot of 2-photon excitation action cross-section at different wavelengths.

Despite the promising applications in imaging, band-shifting photoluminescent materials with 2-photon excitation have been rarely reported [24]. Due to its strong band shift among the CA-based materials under 1P excitation, we further investigated CA-Ser-Urea to characterize its 2PEF properties. The schematic of the experimental setup for the emission property measurement under 2-photon excitation is shown in Fig. 6A. The excitation wavelength scanning was enabled by a wavelength-tunable femtosecond laser (Coherent Chameleon, 80 MHz, 140-fs pulse width). A combination of power control components consisting of a half-wave plate, a polarized beam splitter, and a continuously variable neutral-density filter was used to adjust the laser power reaching the sample. The excitation power before the objective (Mitutoyo Plan APO, 50×, NA 0.55) was measured as a reference since the excitation power inside the sample was not accessible during experiments. The 10 to 20 μl of 10× dilution of as-synthesized CA-Ser-Urea solution was prepared and held in a square miniature hollow glass tubing (VitroTubes, 8290). The epi-detected 2-photon fluorescence was collected by the same 50× objective lens, filtered by a dichroic mirror (Semrock, FF665-Di02) and a low-pass filter (Semrock, FF02-694/SP-25), and focused to a spectrometer (Horiba, iHR320) by an achromatic lens (Edmund Optics, 50 mm). The excitation wavelength was scanned from 700 to 1,000 nm at an interval of 10 nm over the 300-nm scanning range. The emission spectrum at each excitation wavelength was recorded accordingly. For a given excitation wavelength, the 2-photon emission action cross-section of CA-Ser-Urea can be calibrated from the measured 2-photon fluorescence signal, and the fluorescence from a reference sample by using the following Eq. 2 [205]:

$$\sigma_{\mathrm{TPE}A_{s}}=\eta_{2_{s}}\sigma_{2}{\left(\lambda \right)}_{s}=\frac{{\langle F\left(t\right)\rangle }_{s}\phi_{\mathrm{ref}}\eta_{2\;\mathrm{ref}}\sigma_{2}{\left(\lambda \right)}_{\mathrm{ref}}C_{\mathrm{ref}}{\langle P\left(t\right)\rangle }_{\mathrm{ref}}^{2}n_{\mathrm{ref}}}{{\langle F\left(t\right)\rangle }_{\mathrm{ref}}\phi_{s}C_{s}{\langle P\left(t\right)\rangle }_{s}^{2}n_{s}}$$ (2)

where *σ*_TPEA_ is the 2-photon emission action cross-section, *η*_2_ is the quantum efficiency, *σ*_2_ is the 2PCS, 〈*F*(*t*)〉 is time-averaged fluorescence photon counts, *ϕ* is fluorescence collection efficiency, *C* is sample molar concentration, 〈*P*(*t*)〉 is time-averaged excitation power, *n* is sample refractive index, and the subscript “s” and “ref” stand for the sample under study (i.e., CA-Ser-Urea) and the reference sample with a known cross-section. The molar concentration of CA-Ser-Urea is estimated to be 80 mM. Here, we chose 100 μM rhodamine B dissolved in methanol as a reference, and its cross-section at each excitation wavelength and quantum efficiency reported in previous work were used [206]. The spectral response of all optical components in the fluorescence collection setup was considered in the calibration and the refractive index of CA-Ser-Urea is 1.3503.

Two-photon spectra of CA-Ser-Urea at selected 2P excitation wavelengths are shown in Fig. 6B and C. For imaging applications, ideal emission intensity at each excitation wavelength should be high enough and the band shift needs to be pronounced for chromatic imaging. The fluorescence intensities of CA-Ser-Urea produced by all measured excitation wavelengths from 750 to 1,000 nm are of the same order (Fig. 6B), and CA-Ser-Urea shows considerable band shifts, with its fluorescence peak shifting 50 nm when the excitation wavelength changes from 750 to 1,000 nm, as shown in normalized emission spectra in Fig. 6C, both of which could benefit potential chromatic 2-photon fluorescence microscopy applications. To better visualize the spectral shifts or the color changes, the CIE 1931 chromaticity coordinates of CA-Ser-Urea under different wavelengths of 2P excitation are shown in Fig. 6D. Additionally, the fluorescence photon counts with various excitation power are plotted in a logarithmic scale in Fig. 6E. The slopes of all linear fittings for the photon counts and excitation power relationship in logarithmic scale are close to 2, which verifies the quadratic relationship between fluorescence intensity and the excitation laser power and confirms that the fluorescence is 2PEF. The calibrated 2-photon emission action cross-section of CA-Ser-Urea is shown in Fig. 6F. Compared to commercial fluorophores such as rhodamine B, the 2-photon emission action cross-section of CA-Ser-Urea is almost 2 orders smaller, which has plenty of room for improvement. One possible reason is that the active components that result in the band-shifting phenomenon are not fully purified and isolated, and the concentration used for characterization may not accurately reflect the actual concentration of the active components. It is worth mentioning that by comparing the emission spectra or the CIE 1931 coordinates under corresponding excitation wavelengths, we can find that the emissions of CA-Ser-Urea under 1-photon excitation differ from those under 2-photon excitation. For example, under 400-nm excitation, the CIE 1931 coordinates of the emission is (0.186, 0.269), which is cyan-blue color, while under the corresponding 800-nm 2-photon excitation, the CIE 1931 coordinates is (0.312, 0.460), which is green color; under 450-nm 1-photon excitation, the CIE 1931 coordinates is (0.284, 0.499), which is green, while under 900-nm 2-photon excitation, the CIE 1931 coordinates is (0.392, 0.530), which is yellow-green. This indicates that the photophysical processes of CA-Ser-Urea under 1- and 2-photon excitations are different. One possible reason that CA-Ser-Urea exhibits different photoluminescent properties under 1- and 2-photon excitation is to involve different excitation and radiative decay transition pathways. Another hypothetical origin of this difference is the heterogeneity of the emission centers or components inside CA-Ser-Urea sample. In other words, different emission centers/components respond differently to 1- and 2-photon excitation, and the distinct subemissions merge to the distinguishable ensemble emissions under 1- and 2-photon excitations. Since the study is still preliminary, more thorough investigations are needed to reveal the underlying mechanisms in the future. We hope that this discussion will provoke more explorations on 2-photon intrinsic band-shifting materials.

## Potential Applications and Perspective of Citric Acid-Based Intrinsic Band-Shifting Photoluminescent Materials

**Fig. 7.**
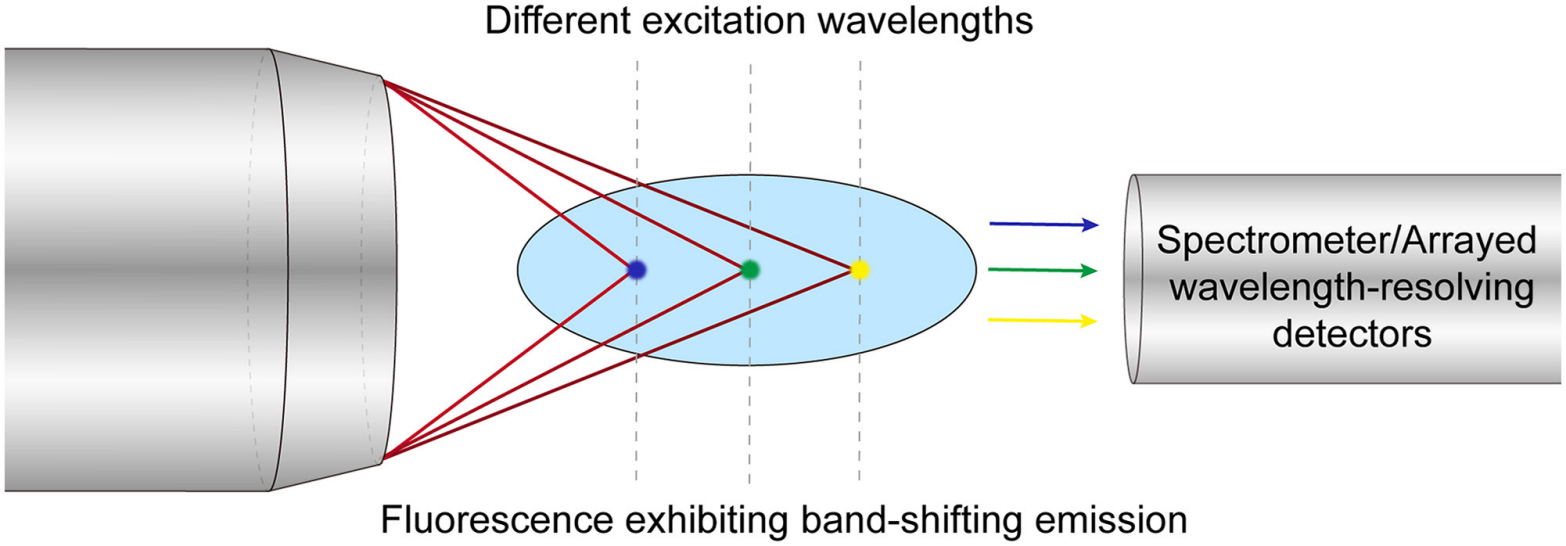
Conceptual schematic of the chromatic fluorescence imaging method. Different excitation wavelengths are focused at different axial positions to excite 1-photon or 2-photon fluorescence exhibiting different band shifts, which are detected in parallel by a spectrometer or wavelength-resolving detectors.

Firstly, the intrinsic band-shifting property of CA-based materials can potentially be exploited in novel imaging applications. In particular, chromatic imaging has been demonstrated as a promising high-speed imaging method, which directs different illumination/excitation wavelengths to different axial locations so as to image a large axial range in parallel. For example, epi-reflection chromatic confocal microscopy maps different wavelengths to different axial positions by purposely introducing chromatic aberration, thus encoding the depth information in the spectral channel and eliminating axial scanning to improve imaging speed [90,207–209]. However, when chromatic imaging is applied to the fluorescence modality, special techniques to resolve the fluorescence emitted from multiple positions are typically required (e.g., by using a micromirror array [210,211]) as the emission spectra of most conventional fluorophores such as organic dyes and inorganic quantum dots remain the same under different excitation wavelengths [174,206]. Therefore, there is still a barrier when chromatic imaging is adapted to fluorescence-based modalities. Previous works have demonstrated the feasibility of applying chromatic imaging to 2-photon fluorescence imaging. For example, a tunable filter in the excitation pathway was utilized to tune the excitation wavelength, which was focused at different depths in a sample by exploiting chromatic aberration to effectively realize axial scanning [91,92]. As the filter can potentially be tuned faster than the mechanical scanning of a sample stage in the axial direction, this helps improve scanning speed. Wavefront separation of the fluorescence signal from different depth levels has also been demonstrated with a micromirror array, where each mirror was conjugated to a specific depth level within the sample [210,211]. In this type of system, the challenge of axial imaging is converted into a lateral one, which can be more easily fulfilled; however, the fluorescence signal decoding relies on a specially designed and fabricated device for wavefront separation. Another way to achieve chromatic imaging without using a wavefront separation device is to apply different fluorophores with various emission wavelengths at different imaging depths, hence encoding the depth information in the spectral channel similar to the epi-reflection chromatic confocal microscopy. However, this requires multiple fluorophores as well as elaborate and cumbersome staining processes. If a single type of fluorophores possesses intrinsic band-shifting property (i.e., the emission wavelengths varying with the excitation wavelength or excitation-wavelength-dependent emissions without the need to tune the working environment), we can avoid the use of multifluorophores and greatly simplify the sample preparation process. Owing to the advantages discussed in Two-photon excited fluorescence properties of CA-Ser-Urea nanoflakes, 2-photon excited fluorescence imaging techniques can have deeper imaging depths and drastically reduce the out-of-focal-plane excitation, therefore increasing the signal-to-noise ratio and mitigating the phototoxicity. By combining chromatic imaging and 2-photon imaging techniques, both high-temporal resolution (i.e., high-speed) and high-spatial resolution imaging can be achieved. A schematic of the proposed chromatic fluorescence imaging using intrinsic band-shifting fluorophores is illustrated in Fig. 7, in which different wavelengths of an excitation source are first focused at different axial positions through purposely introduced chromatic aberration. If the resulting (1- or 2-photon excited) fluorescence generated at different axial positions (thus excited by different wavelengths) exhibit band shifts, it can be imaged in parallel by using a spectrometer (or more generally, arrayed detectors that can resolve different wavelengths, e.g., by using filters). The axial image information is thus encoded in the band-shifting fluorescence spectra for parallel detection.

**Fig. 8.**
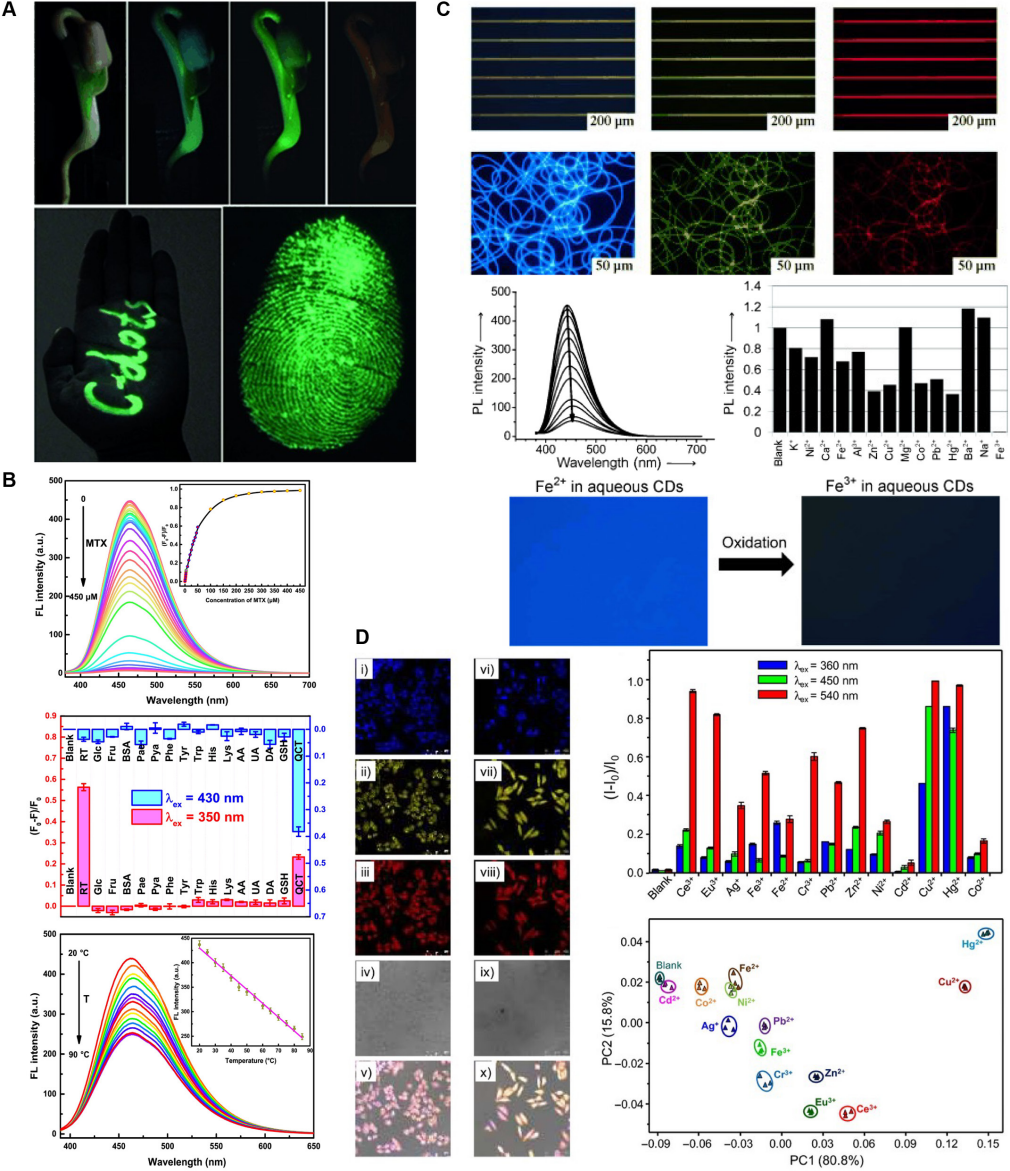
Exemplary applications of CA-based intrinsic band-shifting CDs. (A) Optical and fluorescent images of a bean sprout grown with CDs aqueous solution under daylight, 340-nm excitation, 420-nm excitation, and 500-nm excitation (top), CDs-marked fluorescent characters on human palm captured under 420-nm excitation (bottom left) and a CDs-formed fluorescent fingerprint on commercially available filter paper captured under 420-nm excitation (bottom right). Adapted with permission from [135]. Copyright (2012) Wiley. (B) Fluorescence spectra of the CDs in tris-HCl buffer solution (0.1 mol/l, pH 7.0) upon addition of various concentrations of MTX (from top to bottom: 0 to 450 μM; λ_ex_ = 370 nm and λ_em_ = 460 nm) (top), normalized fluorescence intensities of the CDs solutions (0.5 mg/ml) containing Cu^2+^ (100 μM) in HAc-NaAc buffer solution (0.1 mol/l, pH 5.0) at 450 nm under λ_ex_ = 350 nm in the presence of various chemical compounds at the concentration of 50 μM (red) and at 490 nm under λ_ex_ = 430 nm in the presence of various chemical compounds at the concentration of 10 μM (blue) (middle), and fluorescence spectra of the CDs (0.5 mg/ml) at various temperatures in the range of 20 to 90 °C at 460 nm under λ_ex_ = 370 nm with the inset of the linear relationship between fluorescence intensity and temperature (bottom). Adapted with permission from [131]. Copyright (2018) Elsevier. (C) CDs ink patterned on hydrophilic photoetching stripes and fluorescence microscopy images of polyvinyl alcohol/CDs nanofibers under UV, blue, and green light excitation (top 2), fluorescence quenching in the presence of Fe^3+^ ions (0 to 300 ppm), comparison of fluorescence intensities of CDs after the addition of different metal ions and on/off-switching fluorescence images of aqueous CDs through the oxidation from Fe^2+^ to Fe^3+^ (bottom 2). Adapted with permission from [136]. Copyright (2013) Wiley. (D) Confocal fluorescence images of the F-CDs in MCF-7 cells; i to v) living MCF-7 cells, and vi to x) fixed MCF-7 cells under 405-, 488-, and 543-nm laser excitation, bright field, and merged images, respectively (left), fluorescence responses of the CDs at their 3 emission maxima (i.e., 466, 555, and 637 nm under 360-, 450-, and 540-nm excitation, respectively) to 13 metal ions in Hepes buffer and principal component analysis (PCA) plot for the discrimination of the 13 metal ions based on the triple-channel responses to the CDs (right). Adapted with permission from [128]. Copyright (2015) Wiley. RT, rutin; QCT, quercetin.

Besides imaging applications, due to the tunable emission wavelength, CA-based intrinsic band-shifting materials could be potentially applied in other applications in photonics and optics, for example, working as a novel tunable gain medium to create ultra-broadly tunable laser sources. The intrinsic multicolor emission makes them promising in applications such as display, anticounterfeiting, information encryption, and storage. Additionally, CA-based intrinsic band-shifting materials can be exploited for multimodal sensing if their electronic energy levels exhibit varying sensitivities; in other words, some electronic energy levels are sensitive (or more sensitive) to certain environmental factors or analytes, such as pH, solvent polarity, temperature, pressure, metal ions, and halides, whereas the other electronic energy levels are not. This could allow researchers to achieve multimodal sensing with various excitation wavelengths. Here, multimode means that different analytes can be detected within the same sensing platform. However, if the emission spectra of analytes have a significant overlap or the excitation wavelength chosen is inappropriate when absorption spectra of analytes overlap, the sensitivity and selectivity could be drastically influenced. In contrast to conventional multimodal sensing, intrinsic band-shifting materials can enable excitation wavelength-dependent multimodal sensing by distinguishing different analytes ratiometrically or in other quantitative manners by exploiting the different responses of analytes under various wavelengths of excitation. For example, fluorescence under excitation wavelength 1 (*I*_1_) is sensitive to analyte 1, fluorescence under excitation wavelength 2 (*I*_2_) is sensitive to analyte 2, and fluorescence under excitation wavelength 3 (*I*_3_) is insensitive to both analytes 1 and 2; so, by calculating the *I*_1_/*I*_3_ and *I*_2_/*I*_3_, ratiometric sensing can be achieved and 2 analytes in a single sample can be quantitatively measured simultaneously. It is noted that CA-based small molecular fluorophores, polymers, and CDs all exhibit good biocompatibility and low cytotoxicity, making them an excellent candidate for biomedical or in vivo applications. Qu et al. [135] synthesized a novel type of CA-based CDs and grew a bean sprout in the CDs solution; the bean sprout exhibited band-shifting emission under different wavelengths of excitation, and the bright fluorescence was also demonstrated on a human palm and with a fingerprint on filter paper (Fig. 8A). A type of intrinsic band-shifting CA-based CDs was applied by Li et al. [131] to achieve multimodal sensing with different excitation wavelengths. Three drugs, methotrexate (MTX), rutin, and quercetin, were successfully detected with the assistance of Cu^2+^ in real human urine and human blood samples, and the CDs are also sensitive to temperature change and have been demonstrated in a temperature sensing test (Fig. 8B). Krysmann et al. [137] reported CDs from pyrolysis of CA and ethanolamine exhibiting pH- and metal ion-responsive intrinsic band-shifting properties, which are potentially suitable for the corresponding sensing applications. Another type of CA-derived CDs also exhibited metal ion-sensitive band-shifting emission, especially for Fe^3+^ and on/off fluorescence was further achieved through the oxidation from Fe^2+^ to Fe^3+^ (Fig. 8C) [136]. The CDs ink was also patterned on hydrophilic photoetching stripes, and the CDs were processed with polymers, such as polyvinyl alcohol, affording nanofibers by electrospinning to demonstrate their intrinsic band-shifting emission, good fluorescence stability, and capability for practical applications. Furthermore, Pan et al. [128] reported novel CA-based intrinsic band-shifting CDs working as a multicolor biolabeling reagent in multicolor cellular imaging, and the authors developed a multimodal sensing platform to achieve simultaneous detection and discrimination of diverse metal ions through triple-channel monitoring (466-, 555-, and 637-nm emission maxima under 360-, 450-, and 540-nm excitation, respectively) owing to the different responses to various metal ions (Fig. 8D).

There are still limitations in current CA-based intrinsic band-shifting photoluminescent materials. Firstly, for CA-based small molecular intrinsic band-shifting fluorophores and related polymers, the limited conjugation length and the incompletely conjugated ring structure result in a relatively short emission wavelength. Even with the band-shifting phenomenon, the emission is hardly extended to the NIR region, therefore hindering their applications in vivo or in deep tissue. Furthermore, it also results in a relatively small absorption cross-section or molar extinction coefficient and quantum yield, further hampering their optical utilization. Although the CA-based CDs without band-shifting behaviors could possess higher quantum yields due to TPA type of core structures, the band-shifting CA-based CDs also suffer relatively low quantum yields. The reasons could be attributed to the heterogenicity of the compositions, high nonradiative decay rate from the interactions with solvent or microenvironment and the photophysical natures of CDs, etc. Moreover, the reaction mechanisms, the composition and the origin of the luminescence of CA-based intrinsic band-shifting CDs, even small molecular fluorophores, are still under debate and investigation. Since the mist shrouds these materials, the attempt at improvement or further development has been obstructed. Therefore, the future development directions of CA-based intrinsic band-shifting photoluminescent materials are to (a) thoroughly investigate the structural details via chemical structure characterization methods such as nuclear magnetic resonance spectroscopy, liquid chromatography-mass spectrometry, and infrared spectroscopy, and explore the photophysical origin of the luminescence via synthesis of fluorescent molecules with different structures for fluorescence spectroscopic analysis and theoretical calculations such as density functional theory and time-dependent density functional theory calculations; (b) attempt to extend the emission wavelength to NIR region and improve the absorption cross-section/molar extinction coefficient and quantum yield of CA-based molecular and polymeric intrinsic band-shifting materials through molecular engineering approaches such as introducing new building blocks, like larger conjugated amine-containing compounds, or modifying the current luminophore core structures; (c) increase the quantum yield of CA-based intrinsic band-shifting CDs and reduce their heterogenicity by applying delicate or developing novel and more efficient purification methods or by improving and optimizing the synthetic routes; (d) leverage the intriguing intrinsic band-shifting behaviors in more optical application scenarios, add other functional building blocks or modify the current structures to achieve multimodal imaging, such as combining photoluminescence imaging with photoacoustic imaging, magnetic resonance imaging, computed tomography, etc., and take the advantages of CA, such as good biocompatibility, to develop more biomedical applications.

## Conclusions

CA, a crucial metabolite in most living organisms, exhibits great potential to build excellent biomaterials in plural fields, including photoluminescence imaging. In this review, we summarize recent advances in CA-based intrinsic band-shifting photoluminescent materials, including small molecular fluorophores, polymers and CDs. The fluorescent properties of some representative materials are presented to serve as examples of intrinsic band-shift photoluminescent materials. In particular, a novel CA-based nanomaterial, CA-Ser-Urea, is highlighted due to its appreciable intrinsic band-shifting behavior with a slow quantum yield drop as the excitation wavelength increases and the promising 2-photon excited band-shifting fluorescence with a distinguishable profile from that of its 1-photon excited counterpart. In addition, the potential applications and the future development directions of the intrinsic band-shifting photoluminescent materials are discussed. We hope that this work can shed light on the intrinsic band-shifting phenomenon and lead to more discussions and explorations about band-shifting photoluminescent materials in different fields. We believe that with continuous efforts, the band-shifting materials, especially CA-based intrinsic band-shifting photoluminescent materials, will be applied in vast application scenarios.

## Data Availability

All data are available in the main text.
